# Role of P-Glycoprotein Expression and Function in Cystinotic Renal Proximal Tubular Cells

**DOI:** 10.3390/pharmaceutics3040782

**Published:** 2011-10-27

**Authors:** Karen Peeters, Martijn J. Wilmer, Joost P. Schoeber, Dorien Reijnders, Lambertus P. van den Heuvel, Rosalinde Masereeuw, Elena Levtchenko

**Affiliations:** 1 Pediatric Nephrology, Katholieke Universiteit Leuven, Herestraat 49 Box 817, Leuven, Belgium; 2 Department of Pharmacology and Toxicology (149), Radboud University Nijmegen Medical Center, Geert Grooteplein Noord 21, 6525 GA, Nijmegen, The Netherlands; 3 Department of Pediatrics, Radboud University Nijmegen Medical Center, Geert Grooteplein Zuid 10, 6525 GA, Nijmegen, The Netherlands

**Keywords:** cystinosis, P-glycoprotein, renal proximal tubular cell, cysteamine

## Abstract

P-glycoprotein (P-gp) is an ATP-dependent transporter localized at the apical membrane of the kidney proximal tubules, which plays a role in the efflux of cationic and amphipathic endogenous waste products and xenobiotics, such as drugs, into urine. Studies in mice deficient in P-gp showed generalized proximal tubular dysfunction similar to the phenotype of patients with cystinosis, an autosomal recessive disorder caused by mutations in the lysosomal cystine transporter cystinosin. Renal disease in cystinosis is characterized by generalized dysfunction of the apical proximal tubular influx transporters (so-called renal Fanconi syndrome) developing during infancy and gradually progressing towards end-stage renal disease before the 10th birthday in the majority of patients that are not treated with the cystine-depleting drug cysteamine. Here, we investigated whether the proximal tubular efflux transporter P-gp is affected in cystinosis and whether this might contribute to the development of renal Fanconi syndrome. We used conditionally immortalized (ci) proximal tubular epithelial cells (ciPTEC) derived from cystinotic patients and healthy volunteers. P-gp-mediated transport was measured by using the P-gp substrate calcein-AM in the presence and absence of the P-gp-inhibitor PSC833. P-gp activity was normal in cystinotic cells as compared to controls. Additionally, the effect of cysteamine on P-gp transport activity and phosphate uptake was determined; demonstrating increased P-gp activity in cystinotic cells, and further decrease of proximal tubular phosphate uptake. This observation is compatible with the persistence of renal Fanconi syndrome *in vivo* under cysteamine therapy. In summary, P-gp expression and activity are normal in cystinotic ciPTEC, indicating that P-gp dysfunction is not involved in the pathogenesis of cystinosis.

## 1. Introduction

Cystinosis is an autosomal recessive disorder caused by bi-allelic mutations in the lysosomal cystine transporter cystinosin encoded by the *CTNS* gene [1]. The most common genetic defect in the Northern European population is a large 57 kb deletion, found in up to 60% of the tested alleles [2]. While lysosomal cystine accumulation is a key feature in cystinosis, it is still unclear how cystinosin dysfunction leads to the clinical phenotype of the disease. The renal phenotype of cystinosis is characterized by the development of generalized dysfunction of proximal tubular apical influx transporters (renal Fanconi syndrome) in the majority of patients progressing towards end-stage renal disease in the first 10 years of life. The cystine-depleting drug, cysteamine, postpones the deterioration of renal function. However, renal Fanconi syndrome, when established, is resistant to cysteamine treatment, and remains the main source of morbidity, requiring extensive treatment. Whether proximal tubular efflux transporters are also affected in cystinosis has never been studied.

One of the proximal tubular efflux transporters is P-glycoprotein (P-gp), which is an ATP-dependent transporter localized at the apical plasma membrane, playing a role in the efflux of endogenous waste products and xenobiotics, such as drugs, into urine [3]. Studies in mice genetically deficient in P-gp showed generalized proximal tubular dysfunction (e.g., a two-fold increased urine flow and a loss of sodium, amino acids, low molecular weight proteins, glucose and calcium into the urine) [4], resembling the phenotype of patients with cystinosis. On the other hand, cystinotic ciPTEC has been demonstrated to have decreased intracellular ATP levels [5], which can underlie decreased P-gp activity.

Based on these observations, we hypothesized that P-gp expression and/or function can be affected by cystinosis and that it might contribute to the pathogenesis of renal Fanconi syndrome in this disorder.

Using ciPTEC from cystinosis patients and healthy subjects, we studied the putative role of P-gp in cystinosis by a comprehensive approach including P-gp expression and transporter activity assays, and phosphate uptake, either in the presence or absence of cysteamine treatment.

## 2. Experimental Section

### 2.1. Cell Culture

Conditionally immortalized human proximal tubule epithelial cell lines (ciPTEC) were established and characterized as previously described [5]. Briefly, primary cells were derived from freshly collected urine and conditionally immortalized using SV40T antigen and hTERT vector to maintain proliferation as described in detail by Wilmer *et al.* [6]. In total, clonal ciPTEC were used from 4 healthy controls and 9 cystinosis patients with known mutations in the *CTNS* gene (5 homozygous 57 kb deletion, 4 heterozygous 57 kb deletion combined with other truncating mutations) [5]. All cystinosis patients presented with renal Fanconi syndrome. Routinely, cells were maintained at a permissive temperature of 33 °C (proliferating cells). To decrease expression of SV40T, cells were transferred to 37 °C and allowed to mature for up to 10 days (matured cells). ciPTEC were cultured in DMEM-HAM's F12 medium (Lonza; Basel, Switzerland) supplemented with 10% fetal bovine serum, penicillin/streptomycin (100 U/mL, Gibco), ITS (Insulin 5 μg/mL; transferrin 5 μg/mL and selenium 5 ng/mL, Sigma), epidermal growth factor (10 ng/mL, Sigma), hydrocortisone (36 ng/mL) and tri-iodothyronine (40 pg/mL).

### 2.2. Quantitative PCR (qPCR)

To determine P-gp expression, total RNA from ciPTEC was extracted with TRIzol™ total RNA isolation reagent (Invitrogen, Merelbeke, Belgium) according to the manufacturer's protocol. Residual DNA was removed using DNAse (Invitrogen) according to the manufacturer's instructions. Approximately 1 μg of total RNA, in the presence of RNase I inhibitor (Invitrogen), was used for oligo(dT)-primed first strand cDNA synthesis using M-MLV reverse transcriptase (Invitrogen). qPCR was performed in triplicate using the ABI/PRISM 7900 HT Gene Expression Micro Fluidic Card. β-actin was used as reference gene. In previous studies, we investigated a number of different housekeeping genes in our cell lines and found ß-actin to be most reliable. Primer probe sets for P-gp and β-actin were designed by Applied Biosystems (Lot 714363 and 705007 Applied Biosystems, Zwijndrecht, The Netherlands). The thermal cycling conditions were 2 min at 50 °C and 10 min at 95 °C, followed by 40 cycles of 15 sec at 95 °C and 1 min at 60 °C.

Data are expressed as delta cycle threshold (ΔC_T_) values, indicating the relative P-gp expression levels after correction for β-actin. A Student's t-test was performed to determine the significance of the differences in ΔCt values between patients and controls.

### 2.3. Polyacrylamide Gel Electrophoresis and Western Blotting

For the isolation of membrane vesicles, cell pellets were collected and resuspended in 35 mL ice-cold hypotonic buffer (0.5 mM sodium phosphate, 0.1 mM EDTA; pH 7.0) with freshly added protease inhibitors (phenylmethylsulfonyl fluoride 100 mM, E64 1 mM, aprotinin 1 mg/mL, leupeptin 1 mg/mL and pepstatin 1 mg/mL). Cell suspensions were centrifuged for 30 minutes at 100.000×*g* at 4 °C (Sorvall Ultra WX-80 surespin 630/17 rotor). The supernatant was discarded and pellets were resuspended in 35 mL ice-cold Tris-Sucrose buffer containing 10 mM Tris-Hepes (pH 7.4) and 250 mM sucrose with added protease inhibitors. The solution was homogenized using a tight-fit Douncer (30 strokes) and centrifuged at 500*×*g for 20 minutes at 4 °C (Eppendorf 5804R) to precipitate all cell substances that were not ground by the douncer. Subsequently, the supernatant was centrifuged for 60 min at 100.000×*g*. The vesicle-containing pellet was resuspended in 200 μL Tris-Sucrose buffer and dispersed 30 times through a 27-gauge needle. Protein concentration was determined using the Lowry protein assay method. Expression of P-gp was analyzed in fractions of 10 μg by Western blotting using reduced 6% sodium dodecyl sulphate polyacrylamide gel electrophoreses (SDS-PAGE) and blotting onto a Polyvinylidene Fluoride (PVDF) membrane (Immobilon, Millipore; Bedford, MA, USA). Human kidney homogenate in RIPA buffer (Sigma) was used as positive control.

Membranes were incubated with monoclonal mouse anti-P-gp (1:200 dilution, Abcam) and GAPDH (1:5.000 dilution; Abcam, Cambridge, UK) as a house-keeping antigen, followed by incubation with goat-anti-mouse-HRP conjugate (1:3000 dilution, Dako) and visualization using Pierce ECL Western blotting substrate (Thermo Fisher Scientific, Waltham MA, USA). For semi-quantification, the pixel intensity of the immunospecific bands was measured using ImageJ 1.43u (National Institutes of Health, Bethesda, MD).

### 2.4. Measurement of P-gp Activity

The transport activity of P-gp was determined using a calcein accumulation assay. Cells were cultured to confluency in 96 well plates followed by maturation for 10 days at 37 °C. The experiment was initiated by washing the cells twice in 100 μL Hepes-Tris Buffer (pH 7.4, containing NaCl 132 mM, KCl 0.5 M, CaCl_2_ 1 mM, MgCl_2_ 1 mM, d-Glucose 5.5 mM and HEPES 10 mM). Next, cells were incubated for 60 minutes at 37 °C with 100 μL of the lipophylic non-fluorescent P-gp substrate calcein-AM (1 μM, Invitrogen) in the presence or absence of the P-gp-inhibitor PSC833 (5 μM; kindly provided by Novartis Pharma, Basel, Switzerland) or DMSO vehicle control.

Additionally, the effect of cysteamine on transport activity was investigated. According to pilots in which the optimal concentration and loading time of cysteamine were determined, cells were loaded with cysteamine (1 mM) for 2 h before incubation with calcein-AM in presence of cysteamine in HT-buffer.

Transport activity was stopped by using 100 μL ice-cold HEPES-buffer, followed by cell lysation with 200 μL 0.1% Triton X-100 in Hepes-Tris buffer. Since calcein-AM is intracellularly metabolized into the fluorescent calcein, fluorescence was measured at extinction 485 nm and emission 535 nm using a Victor3 Multiplate Reader (Perkin Elmer Inc.). To this end, 150 μL of each sample was transferred to a white 96 well micro-titer-plate.

P-gp transport activity was determined by calculating the ratio between cellular fluorescence in the presence of PSC833 or cysteamine and the cellular fluorescence measured in their absence (corrected for vehicle). Data are expressed as mean fluorescence ± S.E.M. Mean values were considered to be significantly different when *p* < 0.05 by use of a Student's t-test.

### 2.5. Sodium-Dependent Phosphate Uptake

Phosphate uptake was performed in confluent monolayers of ciPTEC with ^32^PO_4_ (Perkin Elmer, Waltham, Mass., USA) as described before [7]. Cells cultured for 10 days at 37 °C were incubated with 0.2 mM KH_2_PO_4_ (10 μCi/mL) for 5 min in four independent experiments, in the presence of 137 mM sodium salt or 137 mM *N*-methyl-d-glucamine to study sodium-dependent transport. Data are expressed as mean ± S.E.M. Mean values were considered to be significantly different when *p* < 0.05 by use of a Student's t-test.

## 3. Results and Discussion

### 3.1. P-gp Expression

**Figure 1.**
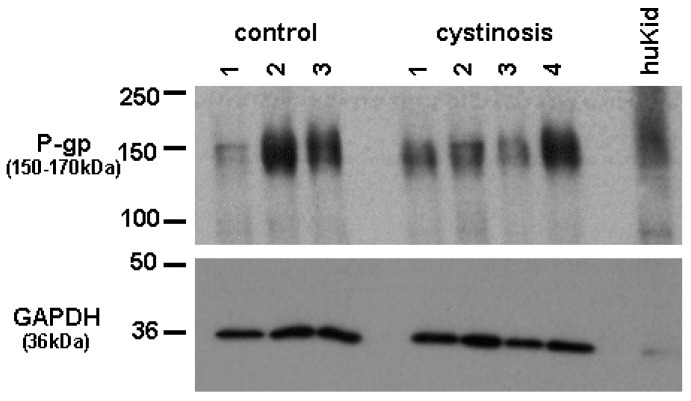
Expression of P-gp (150–170 kDa)) and GAPDH (36 kDa) in membrane vesicles of control and cystinotic ciPTEC and total kidney homogenate (HuKid). Representative blot of two independent measurements is shown.

**Figure 2.**
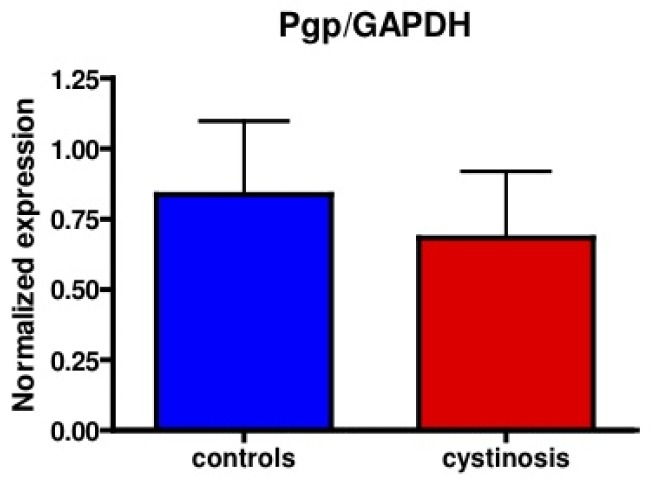
Mean relative protein expression (P-gp/GAPDH) measured by Western blotting in ciPTEC of cystinosis patients (red bar) compared to controls (blue bar). *p* = 0.64.

Total RNA isolation and whole cell homogenates of control and cystinotic cell lines (see Table 1, Figures 1 and 2) were used to study P-gp expression at the gene and protein levels and compared to transporter expression in human kidney. Details of the established controls and cystinotic ciPTEC lines used in this study, including heterozygous *CTNS* mutations, were recently published [5]. P-gp mRNA expression was assessed by qPCR in triplicate for 9 cystinosis patients and the 4 controls, normalized to the average cycle-threshold (C(t)) value for β-actin (C(t) = 18.8 ± 0.8, *n* = 14) and expressed as ΔC(t) (Table 1). Expression of P-gp was similar in cystinosis patients and control cells (*p* = 0.30, 0.29, or 0.15 for the homozygous 57 kb deletion, heterozygous deletion, or total cystinosis population, respectively).

Protein expression of P-gp in cell lines was analyzed using a mouse monoclonal antibody raised against P-gp (C219, purchased from DakoCytomation, the Netherlands). Bands specific for P-gp were detected at approximately 150–170 kDa. Figure 1 shows P-gp and the household protein, GAPDH (36 kDa), expression in isolated membrane vesicles prepared from cystinotic and control ciPTECs, as well as from a total kidney homogenate (Figure 1). Immunospecific bands were semi-quantified by pixel scanning densitometry (Figure 2).

Overall, no statistical difference between cystinotic and control ciPTEC in the levels of P-gp expression was detected.

### 3.2. P-gp Transporter Activity in Cystinotic and Control ciPTEC, at Basal Conditions and after Incubation with Cysteamine

The activity of P-gp was determined in matured confluent ciPTEC of cystinosis patients and controls, by means of the calcein accumulation assay. The selection of cell lines used in these experiments was based on the rate of cell growth, since cell confluency influences P-gp activity [8]. All cells were plated at similar densities at experimental start day -1, cultured overnight at 33 °C, and transferred to 37 °C at day 0, starting maturation. P-gp activity was measured by incubating cells with calcein-AM (1–10 μM) for 60 min at day 7–10 (when the same level of confluency was observed) either in the presence or absence of the P-gp inhibitor PSC833 (5 uM). Intracellular calcein fluorescences were determined.

**Figure 3.**
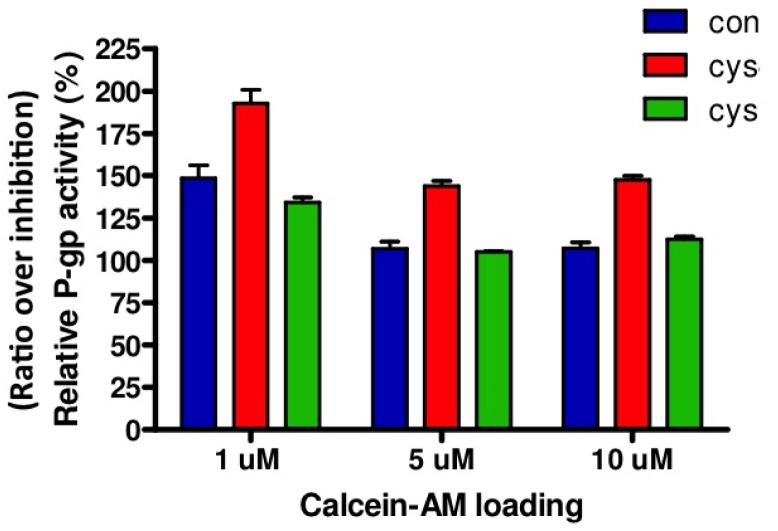
Relative P-gp activity after calcein-AM loading. P-gp activity in two different cystinotic (red and green bar) and one control (blue bar) ciPTEC line was determined after loading the cells with calcein-AM for 60 min (in triplicate) either in the presence of the P-gp inhibitory compound PSC833 (5 μM) or vehicle (0.1% DMSO). Relative P-gp activity is depicted as ratio of P-gp inhibition (PSC833) over the control condition (vehicle). Ratio > 100% indicates inhibition of P-gp activity. Con = control ciPTEC; Cys = cystinotic ciPTEC.

Relative P-gp activity is presented in Figure 3. Both in cystinotic and control ciPTEC, loading of 1 μM calcein-AM resulted in the highest ratio representing P-gp activity. Based on these results, other experiments were conducted with 1 μM calcein-AM. In agreement with unaltered gene and protein expression levels, no differences in P-gp activities were found between cystinotic and control cells.

**Figure 4.**
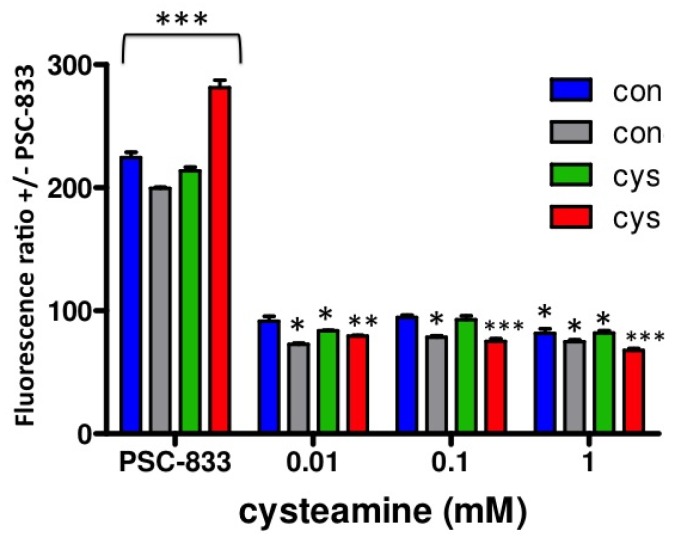
Cysteamine effect on relative P-gp activity. P-gp activity in control (blue and grey bar; n = 2) and in cystinotic ciPTEC (green and red bar; n = 2) was determined after loading the cells with 1 μM calcein-AM for 60 min either in the presence of the P-gp inhibitory compound PSC833 (5 μM), or cysteamine (0.01–1 mM). Relative P-gp activity is depicted as ratio of P-gp inhibition (PSC833) over the control condition (vehicle; 0.1% DMSO). Ratio > 100% indicates inhibition of P-gp activity, whereas a ratio < 100% defines increased P-gp activity. Con = control ciPTEC; Cys = cystinotic ciPTEC. * *p* < 0.05; ** *p* < 0.01; *** *p* < 0.001 *vs.* vehicle treated (100%).

Next, to investigate the putative effects of cysteamine on P-gp activity, we performed a dose-response experiment with cysteamine concentrations ranging from 0.01–10 mM, including 2 h preincubation with identical concentrations. Whereas 0.01–0.1 mM cysteamine are concentrations in the range of those measured in patient's plasma [9], several fold higher concentrations may be expected locally in the kidney tubules due to concentration of the luminal filtrate. As can be observed in Figure 4, similar results were obtained for both control and cystinotic cells treated with 0.01, 0.1 and 1 mM cysteamine, demonstrating that cysteamine slightly, but significantly increases P-gp activity (ratio < 100%; *p* < 0.01). A concentration of 10 mM cysteamine was toxic in all cell lines studied (data not shown). We observed no differences in P-gp activity for the three concentrations of cysteamine tested, suggesting that the maximal effect was already achieved at the lowest concentration used, 0.01 mM cysteamine.

### 3.3. Consequences of P-gp (Dys)function in ciPTEC

**Figure 5.**
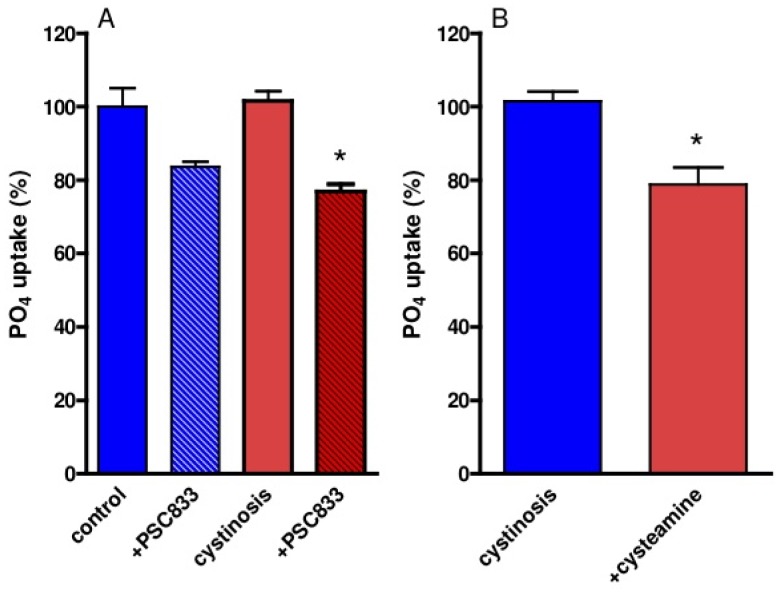
Phosphate uptake in cystinotic and control ciPTEC. (A) Uptake of 0.2 mM ^32^P-labeled phosphate during 5 min in cystinotic (red bars) and control (blue bars) ciPTEC was determined in triplicate with or without preincubation with 5 μM PSC883 or vehicle (0.1% DMSO) for 2 h. (B) Cystinotic cells were preincubated with 1 mM cysteamine (red bar) or vehicle (blue bar) (normal culture medium) for 1 h. Results are presented as % of ^32^PO4 uptake (nmol/μg protein/5 min) *p < 0.05 *vs.* cystinosis.

Sodium-dependent phosphate reabsorption is a differentiated function of renal proximal tubule cells, which was determined in cystinotic and control ciPTEC. Both PSC833 and cysteamine (pre)incubation resulted in decreased uptake of phosphate (Figure 5).

The decreased activity of renal proximal tubular apical influx transporters is a key-feature of the cystinosis phenotype. However, the activity of efflux transporters, such as P-gp, responsible for the excretion of waste products and drugs has not been studied in cystinosis. Based on our previous observation in P-gp deficient mice, developing a renal phenotype resembling that of human cystinosis, we hypothesized that P-gp activity can be disturbed in patients with cystinosis, which in turn can contribute to the pathogenesis of renal Fanconi syndrome. This hypothesis was tested in a recently developed human proximal tubular cell model (ciPTEC) derived from urine of cystinosis patients and healthy controls [5]. Compatible to *in vivo* findings, cystinotic ciPTEC demonstrated decreased sodium-dependant phosphate uptake [5]. However, P-gp expression and activity was comparable between cystinotic and control cells, indicating that P-gp dysfunction is not involved in the pathogenesis of cystinosis.

Interestingly, treatment with the cystine depleting agent cysteamine slightly enhanced P-gp activity in both control and cystinotic ciPTEC. This observation can be of importance for drug dosing in cystinosis patients and should be confirmed *in vivo*, because P-gp is involved in renal clearance of numerous drugs, such as the immunosuppressive calcineurin inhibitors, rapamycin and a variety of cytostatics [10]. Some clinical studies showed better renal graft survival in patients with cystinosis compared to patients with other renal diseases [11,12]. The general belief is that alterations in immune response due to cystine accumulation substantiate this observation [12]. Our results might indicate another potential mechanism, namely reduced degree in calcineurin inhibitors-induced (CNI) toxicity due to the faster elimination of CNI into the proximal tubular lumen by cysteamine mediated P-gp stimulation. Whether the observed small increase in P-gp activity is clinically relevant needs to be determined, but as shown for single nucleotide polymorphisms in MDR1 leading to gain in function of P-gp, as seen for instance for MDR1 polymorphisms in epilepsia treatment [13], this might have an impact for pharmacotherapy. The increase in P-gp activity is interesting and possibly related to multiple binding sites on P-gp that are allosterically linked [14,15].

The decreased phosphate uptake in cystinotic and control ciPTEC after P-gp inhibition is compatible with our earlier observations in a P-gp knock-out mice model [4]. The reason for this effect could be a degree of mitochondrial toxicity because swollen mitochondria and decreased ATP levels were observed in P-gp-deficient mice [4].

Why cystinosis patients treated with cysteamine maintain their renal Fanconi syndrome is unknown. Our previous study demonstrated that cysteamine significantly reduced cystine accumulation in cystinotic ciPTEC and increased their intracellular ATP levels [5]. In this study, counter intuitively, we demonstrate that cysteamine slightly but significantly decreased cellular phosphate uptake. This unexpected observation might underlie the persistence of renal Fanconi syndrome in cystinosis under cysteamine treatment and requires further investigation.

## 4. Conclusions

We demonstrate for the first time that proximal tubular efflux transporter P-gp is intact in the hereditary proximal tubular disorder cystinosis. The cystine depleting agent cysteamine slightly increases P-gp activity, which might be of importance for dosing drugs that are substrates for P-gp in patients with cystinosis on cysteamine treatment.

## Figures and Tables

**Table 1.** P-gp mRNA expression in (ci) proximal tubular epithelial cells (ciPTEC) of cystinosis patients and healthy controls. Data of 13 samples, measured in triplicate, are presented. ΔC(t) values are corrected for the expression of β-Actin, which was used as an internal control (mean C(t) β-Actin: C(t) = 18.1 ± 0.9). β-Actin mRNA expressions were comparable in ciPTEC from controls (C(t) = 18.1 ± 0.7, n = 4) and the three patient populations: homozygous 57 kb deletion C(t) = 18.8 ± 1.2, n = 5; heterozygous 57 deletion combined with another mutation C(t) = 19.2 ± 1.0, n = 4. No differences in P-gp expression between cystinotic and control ciPTEC were observed.

**ciPTEC**	**ΔC_T_ values**	**p-value**
Healthy controls (n = 4)	10.9 ± 3.5	
Patients with homozygous 57kb deletion (n = 5)	9.2 ± 1.2	0.30
Patients with heterozygous 57kb deletion combined with	9.0 ± 1.4	0.35
another mutation (n = 4)		
All cystinosis patients (n = 9)	9.0 ± 1.3	0.15
